# Reproducibility of abdominal fat assessment by ultrasound and computed tomography

**DOI:** 10.1590/0100-3984.2016.0023

**Published:** 2017

**Authors:** Fernando Marum Mauad, Francisco Abaeté Chagas-Neto, Augusto César Garcia Saab Benedeti, Marcello Henrique Nogueira-Barbosa, Valdair Francisco Muglia, Antonio Adilton Oliveira Carneiro, Enrico Mattana Muller, Jorge Elias Junior

**Affiliations:** 1 PhD, Professor and Head of the Graduate Division of the Faculdade de Tecnologia em Saúde (Fatesa), Ribeirão Preto, SP, Brazil.; 2 PhD, Professor in the Department of Radiology at the Universidade de Fortaleza (Unifor), Fortaleza, CE, Brazil.; 3 MSc, Professor at the Faculdade de Tecnologia em Saúde (Fatesa), Ribeirão Preto, SP, Brazil.; 4 Tenured Associate Professor in the Radiology Division of the Department of Clinical Medicine at the Faculdade de Medicina de Ribeirão Preto da Universidade de São Paulo (FMRP-USP), Ribeirão Preto, SP, Brazil.; 5 PhD, Associate Professor in the Radiology Division of the Department of Clinical Medicine at the Faculdade de Medicina de Ribeirão Preto da Universidade de São Paulo (FMRP-USP), Ribeirão Preto, SP, Brazil.; 6 PhD, Physicist, Associate Professor in the Department of Physics and Mathematics at the Faculdade de Filosofia, Ciências e Letras de Ribeirão Preto da Universidade de São Paulo (FFCLRP-USP), Ribeirão Preto, SP, Brazil.; 7 MD, Ultrasound Technician at the Hospital Mãe de Deus, Porto Alegre, RS, Brazil.; 8 Tenured Professor, Coordinator of the Center for Imaging Sciences and Medical Physics of the Faculdade de Medicina de Ribeirão Preto da Universidade de São Paulo (FMRP-USP), Ribeirão Preto, SP, Brazil.

**Keywords:** Abdominal fat, Ultrasonography, Computed tomography, Radiology

## Abstract

**Objective:**

To test the accuracy and reproducibility of ultrasound and computed tomography (CT) for the quantification of abdominal fat in correlation with the anthropometric, clinical, and biochemical assessments.

**Materials and Methods:**

Using ultrasound and CT, we determined the thickness of subcutaneous and intra-abdominal fat in 101 subjects-of whom 39 (38.6%) were men and 62 (61.4%) were women-with a mean age of 66.3 years (60-80 years). The ultrasound data were correlated with the anthropometric, clinical, and biochemical parameters, as well as with the areas measured by abdominal CT.

**Results:**

Intra-abdominal thickness was the variable for which the correlation with the areas of abdominal fat was strongest (i.e., the correlation coefficient was highest). We also tested the reproducibility of ultrasound and CT for the assessment of abdominal fat and found that CT measurements of abdominal fat showed greater reproducibility, having higher intraobserver and interobserver reliability than had the ultrasound measurements. There was a significant correlation between ultrasound and CT, with a correlation coefficient of 0.71.

**Conclusion:**

In the assessment of abdominal fat, the intraobserver and interobserver reliability were greater for CT than for ultrasound, although both methods showed high accuracy and good reproducibility.

## Abstract (pt)

**Objetivo:**

Testar a precisão e a reprodutibilidade da ultrassonografia (US) e da tomografia computadorizada (TC) para a quantificação da gordura abdominal, em correlação com as avaliações antropométricas e clinicolaboratoriais.

**Materiais e Métodos:**

Foram determinadas, por meio da US e da TC, as espessuras subcutâneas e intra-abdominais em 101 indivíduos, sendo 39 homens (38,6%) e 62 mulheres (61,4%), com idade média de 66,3 anos (60-80 anos). Os dados obtidos pela US foram correlacionados com os parâmetros antropométricos, clinicolaboratoriais e com as áreas abdominais medidas pela TC.

**Resultados:**

A espessura da gordura intra-abdominal foi a variável que obteve maior coeficiente de correlação com as áreas adiposas abdominais. A TC mostrou maior reprodutibilidade nas mensurações da gordura abdominal, apresentando maior taxa de concordância intraexaminador e interexaminador em comparação com a US. Observou-se índice de correlação de 71% entre a US e a TC.

**Conclusão:**

Houve maior concordância intraexaminador e interexaminador na avaliação da gordura abdominal por TC, comparativamente à US, porém ambos os métodos mostraram alta precisão e boa reprodutibilidade.

## INTRODUCTION

There is a growing interest in the assessment of body fat distribution, especially for research into metabolic syndrome, obesity and lipodystrophy studies, considering its classifications and prognostic evaluations, as well as follow-up evaluations of dietary and drug treatments. Knowledge about the correlation between body fat distribution and the various anthropometric, biochemical, and functional parameters of anatomical data has increased steadily in recent years^(1-3)^. There is evidence of functional differences between subcutaneous fat and visceral fat; the visualization and quantification of these compartments by imaging methods are useful in building knowledge about these differences^(4)^.

Previous studies have shown the potential of ultrasound and computed tomography (CT) for evaluating the content of abdominal fat^(5-7)^; various other imaging methods, such as radiography, densitometry, and magnetic resonance imaging, have also been used for that purpose^(8-10)^. Ultrasound and CT are both still prominent image methods, mainly due to their wide availability, and well as their relative ease of use and facility in obtaining measurements. It is of note that there is no consensus on which method should be used, mainly due to the difficulty in validating their use in large-scale studies^(10)^.

Given that ultrasound and CT are the main methods used in order to evaluate body fat, especially abdominal fat, the objective of the present study was to evaluate the reproducibility and accuracy of ultrasound as a tool to study abdominal body fat, using CT as the reference. The hypothesis of the study is that, although both methods can be used for assessing abdominal fat, ultrasound would be preferable.

## MATERIALS AND METHODS

This was an observational, cross-sectional study in which abdominal fat was analyzed by anthropometric and imaging methods (ultrasound and CT). The imaging exams were evaluated by two observers who applied identical techniques. The study was approved by the Research Ethics Committee of the Hospital das Clínicas da Faculdade de Medicina de Ribeirão Preto da Universidade de São Paulo (HCFMRP-USP), in the city of Ribeirão Preto, Brazil, and all participating subjects gave written informed consent.

### Selection of subjects

We randomly selected households of individuals residing in the region covered by the Family Health Program of HCFMRP-USP, within a medium- to low-income area with approximately 2000 inhabitants. Subjects between 60 and 80 years of age, living in the selected households, were invited to participate in the study. A total of 112 individuals agreed to participate and were referred to the HCFMRP-USP Department of Geriatrics and Radiodiagnosis. Individuals were selected if they met all of the inclusion and exclusion criteria.

The inclusion criteria were being 60-80 years of age and having cardiovascular disease, diabetes, or another controlled chronic disease. The exclusion criteria were as follows: being bedridden; having thyroid disease; being an alcoholic (consuming ≥ 15 g/day of alcohol) or a smoker; having undergone a surgical procedure, such as liposculpture or liposuction, in the last three months before the evaluation; having any incapacitating disease that would prevent the individual from submitting to the measurements and examinations; and using corticosteroids, anabolic steroids, or hormone replacement therapy.

Anamnesis and physical examination were performed. The study sample included individuals of both genders. Because of the randomized nature of the selection process, individuals were selected without regard to gender, body mass index (BMI), and associated comorbidities, such as diabetes, hypertension, dyslipidemia, and metabolic syndrome. At the time of enrollment, the necessary ancillary examinations were requested. After those evaluations, 11 subjects were excluded because of insufficient data for the statistical analysis. Therefore, the study sample comprised 101 individuals, of whom 39 (38.6%) were men and 62 (61.4%) were women. The mean age of the individuals evaluated was 66.3 ± 4.2 years.

### Clinical, biochemical, and anthropometric data

The presence of comorbidities was evaluated by the staff of the HCFMRP-USP Geriatric Outpatient Clinic. For all subjects, blood pressure was assessed after five minutes of rest. After a 12-hour fast, subjects were submitted to biochemical evaluation to determine their lipid profile (serum levels of total cholesterol, HDL, LDL, and triglycerides). The anthropometric evaluation, according to the criteria of the HCFMRP-USP Metabolic Clinic, included weight (in kg), height (in m), and the calculation of the BMI as weight in kilograms divided by height in meters squared (kg/m^2^). Circumferences (waist, abdomen, and hips) were measured with a tape measure on bare skin.

### Ultrasound evaluation

**Figure 1:**
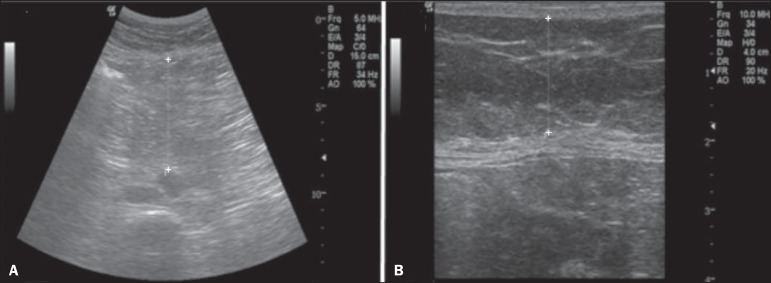
Abdominal ultrasound demonstrating the measurement methods. **A:** Thickness of the visceral fat in the space between the internal (deep) fascia of the rectus abdominis muscle and the anterior wall of the aorta, determined during expiration. **B:** Thickness of the subcutaneous fat in the space between the skin and the external (superficial) fascia of the rectus abdominis muscle.

We quantified the thickness of subcutaneous and visceral abdominal fat (abdominal aorta-wall distance), using a LOGIQ e ultrasound system (General Electric, Milwaukee, WI, USA) with multifrequency convex (3.5-5.0 MHz) and linear (7.5-10.0 MHz) transducers, as depicted in Figure 1.

The thickness of subcutaneous fat was measured with a linear transducer at a frequency of 10.0 MHz. All subjects were evaluated in the dorsal decubitus position, with the right arm elevated, after a 12-hour fast. The transducer was positioned transversely at 1.0 cm above the umbilicus on the xiphoid-pubic line without exerting any pressure on the abdomen, to avoid underestimating the thickness. The anatomical limits for the measurement of subcutaneous fat were the skin and the external (superficial) fascia of the rectus abdominis muscle, and the thickness was quantified in centimeters. The thickness of visceral fat was measured with a convex transducer at a frequency of 4.0 MHz. The transducer was positioned transversely at 1.0 cm above the umbilicus on the xiphoid-pubic line without exerting any pressure on the abdomen, to avoid underestimating the thickness The anatomical limits for the measurement of the visceral fat were the internal (deep) fascia of the rectus abdominis muscle and the anterior wall of the aorta, during expiration, and the thickness was quantified in centimeters.

Ultrasound examinations were performed by two evaluators (specialists in radiology and diagnostic imaging) who used identical examination techniques. Each individual was evaluated on the same day, at different times, and each observer was blinded to the measurements obtained by the other.

### CT evaluation

The subjects underwent CT of the abdomen, from the surface of the diaphragm to the L4-L5 level of the lumbar spine. Scans were acquired with a helical CT scanner (Emotion; Siemens, Erlangen, Germany). The parameters were as follows: thickness, 10.0 mm; interval, 10.0 mm; voltage, 130 kV; current, 250 mA; acquisition time, 6 s; and matrix, 512 × 512 mm. The images were recorded in the supine position, during expiration, and after a 12-hour fast. The superficial fascia defined the division between the superficial and deep subcutaneous fat. The abdominal musculature, in continuity with the deep fascia of the paraspinal muscles, was used in order to distinguish between subcutaneous and visceral fat. The quantity of subcutaneous fat was determined by calculating the difference between the total and visceral fat.

**Figure 2:**
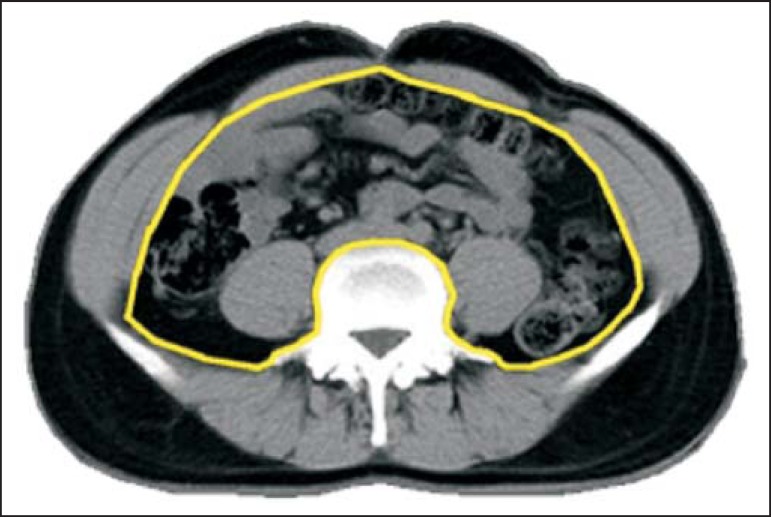
Axial CT scan after manual segmentation of the peritoneal anatomical boundaries and contours of the psoas muscle and vertebral body to determine the area of subcutaneous and visceral fat.

Measurements of the pre-defined areas were obtained by dedicated software developed on the Matlab^®^ platform, version 7.0 (MathWorks, Natick, MA, USA). The area of subcutaneous fat in the L4-L5 slice was calculated by manual segmentation of the appropriate anatomical limits (skin line and external fascia of the muscle), which delimits the entire abdominal area. The sum of the areas of the fat density pixels (between -50 and -250 Hounsfield units) within the limits of the determined area (Figure 2) showed the difference between the total and visceral fat, as previously described^(11)^.

The area of visceral fat was measured in the same slice by manual segmentation of the appropriate anatomical limits (abdominal muscle line, anterior fascia of the psoas muscle, and vertebral body) and the sum of the areas of pixels with a density between -50 and -250 Hounsfield units, within the limits of the determined area (Figure 2). Each observer took two measurements.

### Statistical analysis

Statistical analysis was performed using the Statistical Package for the Social Sciences, version 16.0 (SPSS Inc., Chicago, IL, USA). The reproducibility of the measurements was evaluated by determining the intraclass correlation coefficients and the 95% confidence intervals.

For the determination of intraobserver reproducibility, we considered the values of the two measurements made only by the first observer; for the determination of interobserver reproducibility, we considered only the first measurement made by each observer for each parameter.

When the null hypothesis was rejected, the nonparametric Mann-Whitney test was used. When normality was accepted, the Student’s t-test for independent samples was used. Student’s t-tests, Pearson’s correlation coefficient, and multiple regression analysis were used as appropriate. In the calculation of the Pearson’s correlation coefficients, all correlations higher than 0.20 or lower than -0.20 were considered significant. For all statistical analyses, the level of significance was set at *p* ≤ 0.05.

## RESULTS

Of the 101 individuals who participated in the study, 39 (38.6%) were men and 62 (61.4%) were women. Tables 1 through 4 show the data collected for the 101 subjects evaluated. The mean values obtained by ultrasound, with their respective standard deviations, were 1.88 cm ± 0.97 for the subcutaneous fat thickness and 6.27 cm ± 2.34 for the visceral fat thickness (Table 3). The mean values obtained by CT, with their respective standard deviations, were 635.7 ± 151.1 cm^2^ for the total abdominal area, 396.6 ± 141.8 cm^2^ for the total area of abdominal fat, 220.8 ± 75.0 cm^2^ for the intra-abdominal area, and 136.3 ± 62.9 cm^2^ for the area of intra-abdominal fat (Table 4).

**Table 1:** Distribution of means and standard deviations, with minimum and maximum values for age and anthropometric data.

Variable	Mean	SD	Minimum	Maximum
Age (years)	66.3	4.3	61	80
Height (cm)	159.3	9.5	139.5	182.5
Weight (kg)	70.0	11.9	38.9	97.4
Waist circumference (cm)	89.8	9.7	63	113
Hip circumference (cm)	101.3	8.5	83.2	127
Abdominal circumference (cm)	94.8	11.1	66.5	119.8
Body mass index (kg/m2)	27.6	4.3	17.5	37.4

SD, standard deviation.

**Table 3:** Distribution of means and standard deviations, with minimum and maximum values, for the CT measurements, in cm^2^.

Variable	Mean	SD	Minimum	Máximum
CT-IAA1a	220.8	74.9	77	476
CT-IAA1b	222.9	75	80	475
CT-IAA2	222.9	75.5	75	477
CT-IAFA1a	136.3	62.9	30	390
CT-IAFA1b	137.1	63.2	27	390
CT-IAFA2	137.4	63.6	28	391
CT-AA1a	635.7	151.1	295.8	970.8
CT-AA1b	636.8	151.4	296.6	972.5
CT-AA2	636.4	150.8	295.1	965.9
CT-AFA1a	396.6	141.8	81.2	748.8
CT-AFA1b	396.8	141.3	82.1	747.9
CT-AFA2	393.7	141.4	81.8	738.8

SD, standard deviation; CT-IAA1a, CT of the intra-abdominal area, observer 1 (1st measurement); CT-IAA1b, CT of the intra-abdominal area, observer 1 (2nd measurement); CT-IAA2, CT of the intra-abdominal area, observer 2 (1st measurement); CT-IAFA1a, CT of the intra-abdominal fat area, observer 1 (1st measurement); CT-IAFA1b, CT of the intra-abdominal fat area, observer 1 (2nd measurement); CT-IAFA2, CT of the intra-abdominal fat area, observer 2 (1st measurement); CT-AA1a, CT of the abdominal area, observer 1 (1st measurement); CT-AA1b, CT of the abdominal area, observer 1 (2nd measurement); CTAA2, CT of the abdominal area, observer 2 (1st measurement); CT-AFA1a, CT of the abdominal fat area, observer 1 (1st measurement); CT-AFA1b, CT of the abdominal fat area, observer 1 (2nd measurement); CT-AFA2, CT of the abdominal fat area, observer 2 (1st measurement).

**Table 4:** Reproducibility of the method, as determined by the intraobserver and interobserver intraclass correlation coefficients.

Variable	ICC	95% CI
US-SC1a/US-SC1b	0.946	0.992-0.996
US-SC1a/US-SC2	0.940	0.982-0.992
US-VISC1a/US-VISC1b	0.995	0.993-0.997
US-VISC1a/US-VISC2	0.983	0.976-0.989
CT-IAA1a/CT-IAA1b	0.998	0.997-0.999
CT-IAA1a/CT-IAA2	0.998	0.997-0.999
CT-IAFA1a/CT-IAFA1b	0.999	0.999-0.999
CT-IAFA1a/CT-IAFA2	0.999	0.998-0.999
CT-AA1a/CT-AA1b	1.00	1.000-1.000
CT-AA1a/CT-AA2	0.999	0.999-0.999
CT-AFA1a/CT-AFA1b	1.00	1.000-1.000
CT-AFA1a/CT-AFA2	0.998	0.999-0.999

ICC, intraclass correlation coefficient; 95% CI, 95% confidence interval; US-SC1a, ultrasound of subcutaneous fat, observer 1 (1st measurement); US-SC1b, ultrasound of subcutaneous fat, observer 1 (2nd measurement); US-SC2, ultrasound of subcutaneous fat, observer 2 (1st measurement); US-VISC1a, ultrasound of visceral fat, observer 1 (1st measurement); US-VISC1b, ultrasound of visceral fat, observer 1 (2nd measurement); US-VISC2, ultrasound of visceral fat, observer 2 (1st measurement); CT-IAA1a, CT of the intra-abdominal area, observer 1 (1st measurement); CT-IAA1b, CT of the intra-abdominal area, observer 1 (2nd measurement); CT-IAA2, CT of the intra-abdominal area, observer 2 (1st measurement); CT-IAFA1a, CT of the intra-abdominal fat area, observer 1 (1st measurement); CT-IAFA1b, CT of the intra-abdominal fat area, observer 1 (2nd measurement); CT-IAFA2, CT of the intra-abdominal fat area, observer 2 (1st measurement); CT-AA1a, CT of the abdominal area, observer 1 (1st measurement); CT-AA1b, CT of the abdominal area, observer 1 (2nd measurement); CT-AA2, CT of the abdominal area, observer 2 (1st measurement); CT-AFA1a, CT of the abdominal fat area, observer 1 (1st measurement); CT-AFA1b, CT of the abdominal fat area, observer 1 (2nd measurement); CT-AFA2, CT of the abdominal fat area, observer 2 (1st measurement).

The differences between the subcutaneous and visceral fat measurements did not differ significantly between the two evaluators or between two measurements of a given parameter made by the same evaluator (*p* < 0.001 and *p* = 0.001, respectively), indicating that the results did not differ among themselves in terms of the measurements, whether obtained by ultrasound or by CT.

The associations that the anthropometric data present with the first ultrasound and CT measurements made by the same examiner were studied by calculating the Pearson’s correlation coefficients (Tables 5 and 6).

**Table 5:** Evaluation of the associations between clinical/anthropometric measures and the measures of abdominal fat obtained by CT and ultrasound, as determined by the same observer.

	Correlations*								
	Body mass			Total	Systolic blood	Diastolic blood			Abdominal
	index	Triglycerides	HDL	cholesterol	pressure	pressure	Waist	Hip	circumference
Body mass index	1.00	0.21	-0.14	-0.21	0.40	0.43	0.66	0.70	0.71
Tryglicerides	0.21	1.00	-0.1	0.37	0.16	0.21	0.34	0.35	0.36
HDL	-0.1	-0.1	1.00	0.61	-0.20	-0.1	-0.23	-0.22	-0.21
Total cholesterol	-0.2	0.37	0.61	1.00	-0.42	0.23	0.001	0.01	0.019
Systolic blood pressure	0.39	0.16	-0.20	-0.42	1.00	0.86	0.40	0.40	0.41
Diastolic blood pressure	0.43	0.21	-0.16	0.23	0.86	1.00	0.36	0.37	0.42
Waist	0.66	0.34	-0.22	0.10	0.40	0.36	1.00	0.52	0.94
Hip	0.68	0.34	-0.20	0.12	0.40	0.41	0.52	1.00	0.62
Abdominal circumference	0.71	0.35	-0.21	0.19	0.41	0.42	0.94	0.62	1.00
US-SC1a	0.49	-0.3	-0.20	-0.22	0.27	0.31	0.18	0.47	0.25
US-VISC1a	0.61	0.27	-0.22	0.75	0.30	0.29	0.65	0.51	0.73
CT-IAA1a	0.39	0.27	-0.11	0.48	0.18	0.20	0.62	0.30	0.66
CT-IAFA1a	0.47	0.32	-0.12	0.71	0.22	0.25	0.66	0.38	0.71
CT-AA1a	0.82	0.25	-0.10	0.62	0.42	0.43	0.77	0.79	0.84
CT-AFA1a	0.82	0.18	-0.03	0.75	0.42	0.46	0.65	0.81	0.73

* Values above 0.20 or below -0.20 considered significant.

US-SC1a, ultrasound of subcutaneous fat, observer 1 (1st measurement); US-VISC1a, ultrasound of visceral fat, observer 1 (1st measurement); CT-IAA1a, CT of the intra-abdominal area, observer 1 (1st measurement); CT-IAFA1a, CT of the intra-abdominal fat area, observer 1 (1st measurement); CT-AA1a, CT of the abdominal area, observer 1 (1st measurement); CT-AFA1a, CT of the abdominal fat area, observer 1 (1st measurement).

**Table 6:** Evaluation of the associations between CT and ultrasound measurements obtained by the same observer.

	Correlations					
	US-SC1a	US-VISC1a	CT-IAA1a	CT-IAFA1a	CT- AA1a	CT-AFA1a
US-SC1a	1.00	0.20	-0.18	-0.06	0.31	0.42
US-VISC1a	0.20	1.00	0.68	0.71	0.62	0.65
CT-IAA1a	-0.18	0.68	1.00	0.95	0.67	0.51
CT-IAFA1a	-0.06	0.71	0.95	1.00	0.73	0.64
CT-AA1a	0.31	0.74	0.67	0.73	1.00	0.94
CT-AFA1a	0.42	0.65	0.51	0.64	0.94	1.00

US-SC1a, ultrasound of subcutaneous fat, observer 1 (1st measurement); US-VISC1a, ultrasound of visceral fat, observer 1 (1st measurement); CT-IAA1a, CT of the intra-abdominal area, observer 1 (1st measurement); CT-IAFA1a, CT of the intra-abdominal fat area, observer 1 (1st measurement); CT-AA1a, CT of the abdominal area, observer 1 (1st measurement); CT-AFA1a, CT of the abdominal fat area, observer 1 (1st measurement).

## DISCUSSION

Ultrasound was proposed as an alternative for the evaluation of abdominal adiposity, because of its good correlation with the quantity of visceral fat determined by CT, as well as because it is a noninvasive, practical, effective, low-cost and radiation-free method. To determine the reliability and reproducibility of ultrasound, in terms of the quantification of these fat volumes, we initially used measurements made in healthy individuals, with anthropometric indices that we believe represent the average Brazilian population over 60 years of age, showing an intraobserver difference lower than 12.0% for the ultrasound measurement of subcutaneous fat and lower than 8.0% for the ultrasound measurement of visceral fat. The interobserver difference was lower than 17.6% and lower than 14.0%, respectively, for the ultrasound measurement of subcutaneous and visceral fat.

The results of the present study indicate that ultrasound shows high reproducibility, with intraobserver and interobserver agreement rates of 0.94 and 0.94, respectively, for the ultrasound measurement of subcutaneous fat, compared with 0.99 and 0.98, respectively, for the ultrasound measurement of visceral fat. These findings are in agreement with those of various other studies^(8,11-14)^. Despite differences in methodology and in the statistical analysis, all of those studies reported high reproducibility, with intraobserver and interobserver coefficients of variation lower than 6.5% and lower than 7%, respectively. A recent analysis of interobserver reproducibility found the intraclass correlation coefficient for the measurement of subcutaneous fat and visceral fat to be 0.97 (95% confidence interval: 0.96-0.99; *p* < 0.01) and 0.91 (95% confidence interval: 0.86-0.95; *p* < 0.01), respectively^(4)^. There is a tendency in the literature to propose that ultrasound be used as a routine screening method in the evaluation of visceral fat and repeated as necessary^(2,8)^. However, some studies have shown that its reproducibility and objectivity in such evaluations are highly debatable, because it is a method that is highly dependent on the skill of the operator^(12)^.

Ultrasound offers a simple technique of abdominal fat measurement, which requires only the use of a 3.5-MHz transducer placed 1 cm from the umbilicus, which corresponds to the same area analyzed by CT. The technique was proposed in the 1990s by Armellini et al.^(15)^, who compared ultrasound with CT in a sample of 50 obese women. In that study, the authors found that ultrasound showed a good correlation with CT (*r* = 0.66, *p* < 0.001), inferring, from their research, the applicability of ultrasound in the evaluation of abdominal fat, as demonstrated in a subsequent study^(12)^. In the present study, the thickness of the visceral fat measured by ultrasound correlated well with the area quantified by CT (*r* = 0.71, *p* < 0.001). In addition, using ultrasound, we were able to visualize and measure the “distances” of the subcutaneous and visceral abdominal fat separately.

CT is considered the best imaging method for assessing body components because of its reproducibility and because the fat mass thus obtained has shown correlation coefficients above 0.90 when compared with the actual amount present in a cadaver^(3)^. CT facilitates the differentiation between the subcutaneous and visceral adipose tissue compartments^(1)^.

The choice to perform the scanning in the umbilical region was initially proposed by Borkan et al.^(7)^, who showed that it is a site with a high percentage of body fat and that allows better differentiation between subcutaneous tissue and intra-abdominal fat. Subsequently, Kvist et al.^(16)^ demonstrated that the L4-L5 level presents the best and highest correlation with abdominal fat in both genders. Another study showed that the area of visceral fat measured in a single CT slice, at the level of the umbilicus (L4-L5), correlates strongly with the total volume of visceral fat, which supports the use of this method for the diagnosis of visceral fat deposition^(17)^. In that study, the reliability and reproducibility of the method was verified, because it demonstrated a maximum intraobserver variation of 1.0%, 1.1%, 5.5%, and 5.0%, respectively, between CT measurements of the abdominal area, abdominal fat area, intra-abdominal area, and intra-abdominal fat area, compared with 2.1%, 5.1%, 5.7%, and 6.0%, respectively, for the maximum interobserver variation^(17)^. That indicates that CT can be considered the gold standard, not only for assessing abdominal fat tissue but also for measuring multiple compartments of the body^(17)^. Chowdhury et al.^(18)^ showed that CT has high reproducibility by analyzing 28 scans to determine the intraobserver reproducibility of abdominal fat quantification by CT, showing an intraobserver variability of 0.4% for abdominal fat, compared with 1.0% for the interobserver variability.

In the present study, we evaluated how well ultrasound and CT data correlated with anthropometric data. We found that the CT data showed a stronger correlation with the anthropometric data than did the ultrasound data. That result allows us to state that CT is more appropriate than is ultrasound for the study of abdominal fat in the characterization of individual body composition, given that it shows a greater correlation with the various anthropometric parameters evaluated, as evidenced by the Pearson’s correlation coefficients of 0.82, 0.77, 0.79, and 0.84, respectively, for BMI, waist circumference, hip circumference, and abdominal circumference. This leads us to agree with several authors who consider CT the reference for the evaluation of body fat^(8,11-15,19-21)^. However, it is necessary to consider the risks of the use of ionizing radiation for an evaluation whose clinical significance is relative; that is, although CT provides greater reproducibility and correlates better with the anthropometric data, its use does not seem justifiable in view of the individual risk-benefit ratio^(22)^.

The results obtained, together with the advantages of the ultrasound method, including the fact that it is a rapid, easy to use method with good specificity and reproducibility, suggest that ultrasound is a potentially useful option for the study of visceral obesity in patients at high risk for metabolic syndrome, despite presenting lower reproducibility and a weaker correlation with anthropometric data than does CT. In the present study, we also found that ultrasound showed an excellent correlation with certain measures, mainly BMI, serum cholesterol, and waist circumference. Other authors have demonstrated an excellent correlation between ultrasound and anthropometric measures, stressing the advantages of it being less costly than CT or MRI and more accurate than direct anthropometric measurements^(13,14)^.

## CONCLUSION

In the evaluation of abdominal fat, ultrasound showed good intraobserver and interobserver agreement, as did CT to a greater degree. Therefore, both methods present acceptable accuracy and good reproducibility. Using CT as the reference, we observed a correlation with ultrasound assessments of abdominal fat. CT correlated better with anthropometric measures than did ultrasound, mainly for the measurement of total abdominal fat. Among the clinical and biochemical data, CT and ultrasound both correlated well with the serum cholesterol level.

## Figures and Tables

**Table 2:** Distribution of means and standard deviations, with minimum and maximum values, for the ultrasound measurements, in mm.

Variable	Mean	SD	Minimum	Maximum
US-SC1a	18.8	9.4	5.3	57.9
US-SC1b	18.9	9.3	5.3	57.9
US-SC2	18.4	9.5	3	57
US-VISC1a	62.7	23.4	19.6	130
US-VISC1b	63.1	23.3	18.7	132
US-VISC2	62.5	23.9	19.2	132

SD, standard deviation; US-SC1a, ultrasound of subcutaneous fat, observer 1 (1st measurement); US-SC1b, ultrasound of subcutaneous fat, observer 1 (2nd measurement); US-SC2, ultrasound of subcutaneous fat, observer 2 (1st measurement); US-VISC1a, ultrasound of visceral fat, observer 1 (1st measurement); US-VISC1b, ultrasound of visceral fat, observer 1 (2nd measurement); US-VISC2, ultrasound of visceral fat, observer 2 (1st measurement).
